# Supplementary material for: The interactome of intact mitochondria by cross-linking mass spectrometry provides evidence for coexisting respiratory supercomplexes
Source: Mol Cell Proteomics. 2017 Dec 8;17(2):216–32. doi: 10.1074/mcp.RA117.000470 (PMC5795388; doi:10.1074/mcp.RA117.000470)
Supplement: Supplemental Data [file supp_RA117.000470_133922_0_supp_23978_fzffwf.zip › spectra_annotation/mito_DR_spectra_annotation/2-1-6-1-13-1.pdf]

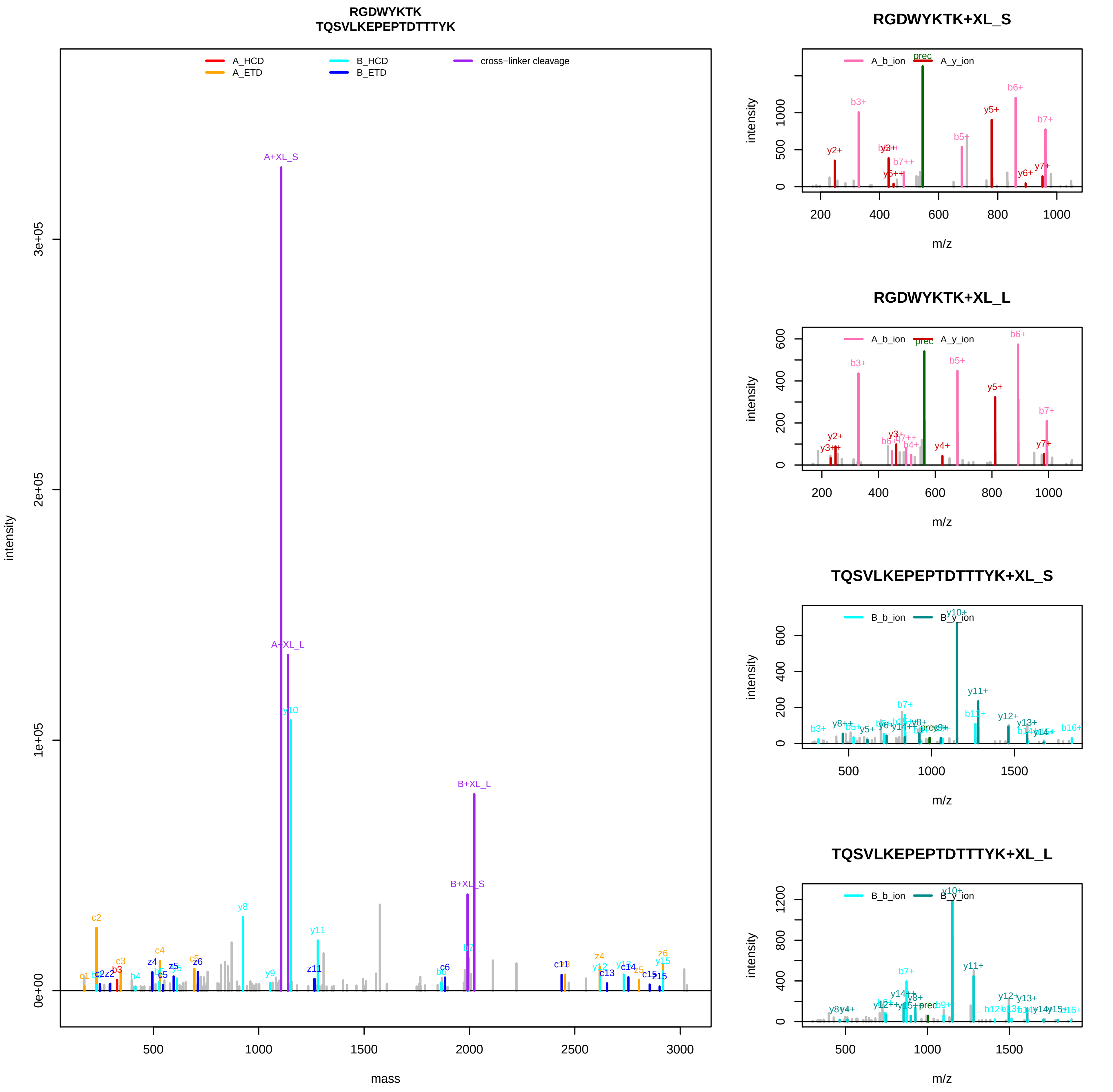

RGDWYKTK
TQSVLKEPEPTDTTTYK
A_HCD
A_ETD
B_HCD
B_ETD
cross−linker cleavage
A+XL_S
A+XL_L
B+XL_L
B+XL_S
y10
y8
y11
y9
z11
c2
c3
c4
c5
b3
z4
z5
z6
c6
b7
c11
c13
c14
c15
z15
y12
y13
y15
z4
z5
z6
intensity
mass
0e+00
1e+05
2e+05
3e+05
500
1000
1500
2000
2500
3000
RGDWYKTK+XL_S
RGDWYKTK+XL_L
TQSVLKEPEPTDTTTYK+XL_S
TQSVLKEPEPTDTTTYK+XL_L
A_b_ion
A_y_ion
B_b_ion
B_y_ion
prec
b3+
b5+
b6+
b7+
b7++
y2+
y3+
y5+
y6+
y7+
y4+
y10+
y11+
y12+
y13+
b16+
m/z
